# An Observational Comparative Study of the Impact of Accidental Pneumoperitoneum in Understanding the Learning Curve for Totally Extraperitoneal and Extended Totally Extraperitoneal Inguinal Hernia Repair

**DOI:** 10.7759/cureus.40013

**Published:** 2023-06-05

**Authors:** Rajeev Bilaskar, Santosh Thorat, Balaji Dhaigude

**Affiliations:** 1 Department of General Surgery, Pimpri Chinchwad Municipal Corporation’s Postgraduate Institute (PCMC's PGI) Yashwantrao Chavan Memorial (YCM) Hospital, Pune, IND

**Keywords:** accidental pneumoperitoneum, inguinal hernia, totally extraperitoneal repair, extended totally extraperitoneal repair, minimally invasive

## Abstract

Background: Abdominal wall hernia repair is among the fundamental procedures in general surgical practice. Subsequent to the advent of minimally invasive repair, there have been efforts to find the most reliable technique, with easily reproducible results that can be practiced by surgeons worldwide. From an analytical point of view, this study aimed to highlight the advantages and disadvantages of two techniques.

Materials and methods: A total of 60 participants were divided into two groups of 30 patients: the totally extraperitoneal (TEP) and the extended TEP (eTEP) hernia repair groups. Covariates and outcomes were analyzed using the chi-square and Mann-Whitney U tests. The study was carried out at a tertiary postgraduate teaching hospital in the western zone of Maharashtra, Pune, India, by a single surgeon. The operative procedures were as per standard surgical practice for both groups. The study was conducted to understand types of difficulties observed in the early implantation stages and the learning curve of these procedures.

Results: Ten percent of the procedures in the TEP group and 6.7% of procedures in the eTEP group required Veress needle use to manage accidental pneumoperitoneum (P=0.64). The mean operative time in the eTEP group was significantly shorter than that of the TEP group (P=0.031).

Conclusion: Compared with the TEP approach, eTEP repair is associated with shorter operative times, owing to a shorter learning curve, a wider-angle view, a wider range of motion for instrument manipulation, and an ergonomically superior operative experience.

## Introduction

Laparoscopic hernia repair is relatively new modality for minimally invasive hernia repair, with the endoscopic extraperitoneal approach preferred over the transabdominal preperitoneal (TAPP) approach. The major advantage of the former is that it does not involve entry into the abdominal cavity, thus lowering the risk of intestinal and vascular injuries as well as herniation at trocar sites [1]. 

Totally extraperitoneal (TEP) repair is a minimally invasive inguinal hernia repair technique in which the preperitoneal space (below the rectus muscle and above the peritoneum) is approached via the anterior abdominal wall without breaching the peritoneum. This technique differs from TAPP repair, wherein the same space is approached by raising a peritoneal flap intraabdominally at the cost of putting the intraabdominal organs at risk of injury.

Extended or enhanced TEP (eTEP) repair is similar to TEP repair, but it allows a better view and more space for meticulous dissection due to flexible port positions, crossover at the midline, and distal placement of the camera port. It adds better surgical ergonomics to the classic TEP approach, thus shortening the learning curve for early-career surgeons.

The extraperitoneal approach is based on Rives-Stoppa technique. The classical TEP technique described by Phillips and McKenna in 1990 has several drawbacks, including limited space for dissection and mesh placement, restricted port placement, possible intolerance of pneumoperitoneum, and difficulty in teaching and learning the technique. Thus, in 2012, Prof. Jorge Daes introduced the eTEP technique for inguinoscrotal hernia repair. The eTEP technique ensures that the extraperitoneal space can be reached from almost anywhere in the anterior abdominal wall.

The eTEP approach quickly and easily creates an extraperitoneal space, enlarges the surgical field, provides a flexible port setup adaptable to many situations, allows unencumbered dissection of the cord structures (proximal dissection of the sac and peritoneum), and facilitates management of the distal sac [2]. In this study we compared the TEP and eTEP techniques at the early stages of the surgical learning curve.

Surgical learning curves involve both subjective and objective factors. Operative time, visceral injuries, vascular injuries, and number of cases to report no complications are some of the objective factors. In this study, we aimed to highlight accidental infliction of peritoneal rents (causing pneumoperitoneum) as an objective variable to understand the learning curves associated with both techniques. Peritoneal rents may result from difficulties in obtaining a clear surgical view, a paucity of dissection space, or a lighter plane of anesthesia wherein diaphragmatic contraction causes a rent in the peritoneum or expands a preexisting rent.

## Materials and methods

Objectives

The primary aim was to compare incidence of accidental pneumoperitoneum associated with TEP vs. eTEP repair in the early learning phases of surgeons performing these procedures. Other perioperative variables were also analyzed for statistical significance.

As secondary objectives, we aimed to compare the two minimally invasive approaches for inguinal hernia surgery with respect to: duration of surgery, duration of hospital stay, time to return to daily activities, intraoperative complications (i.e., visceral injuries and vascular injuries), postoperative pain, and postoperative complications.

Study design

This was a prospective, observational study conducted in Pune, Maharashtra, India. The analysis included 60 patients who were diagnosed with inguinal hernia in a general surgery outpatient department and were randomized to undergo elective inguinal hernia repair with either the laparoscopic TEP or eTEP technique between February 2021 and June 2022. All the procedures were performed by a single surgeon at a single institute at the level of associate professor.

Inclusion and exclusion criteria

Patients (aged 18 years or older) with direct and/or indirect inguinal hernias were eligible for inclusion. We excluded patients with complicated hernias, such as obstructed, incarcerated, or strangulated hernias. We also excluded cases in which general anesthesia was contraindicated.

Sample size

The sample size was calculated with the assumption that 50% of TEP patients and 10% of eTEP patients would develop pneumoperitoneum [3,4]. The main consideration contributing to these assumptions was the longer learning curve associated with TEP over eTEP repair.

Formula

The following formula was used to calculate the sample size:

n = (Zα/2+Zβ)2 * (p1(1-p1) + p2(1-p2)) / (p1-p2)2,

Zα/2 is the critical value of the normal distribution at α/2 (e.g., at a 95% confidence level, α is 0.05, and the critical value is 1.96).

Zβ is the critical value of the normal distribution at β (e.g., for a power of 90%, β is 0.1, and the critical value is 0.84)

p1=50% and p2=10% are the expected sample proportions of the TEP and eTEP groups, respectively.

The minimum required sample size per group was determined to be 23. Allowing for a 10% dropout rate, the minimum required sample size was 26 per group. In the end, 30 participants per group were enrolled.

Procedures

We obtained written informed consent from all participants before their enrollment into the study, which was conducted according to Pimpri Chinchwad Municipal Corporation’s Postgraduate Institute Yashwantrao Chavan Memorial Hospital's institutional ethics committee’s guidelines (registration no. ECR/1236/INST/MH/2019). Patients were enrolled as per the inclusion criteria, and the participants were randomly allocated into two groups using block randomization (creating blocks of four participants each) with an allocation ratio of 1:1 for each group.

All patients underwent thorough history taking and physical examination. Preanesthetic evaluations and other relevant investigations were conducted. The operative procedure were as per standard surgical practice for the two techniques. All patients were followed-up at three, six, and 12 postoperative months.

Operative steps for TEP 

**Figure 1:**
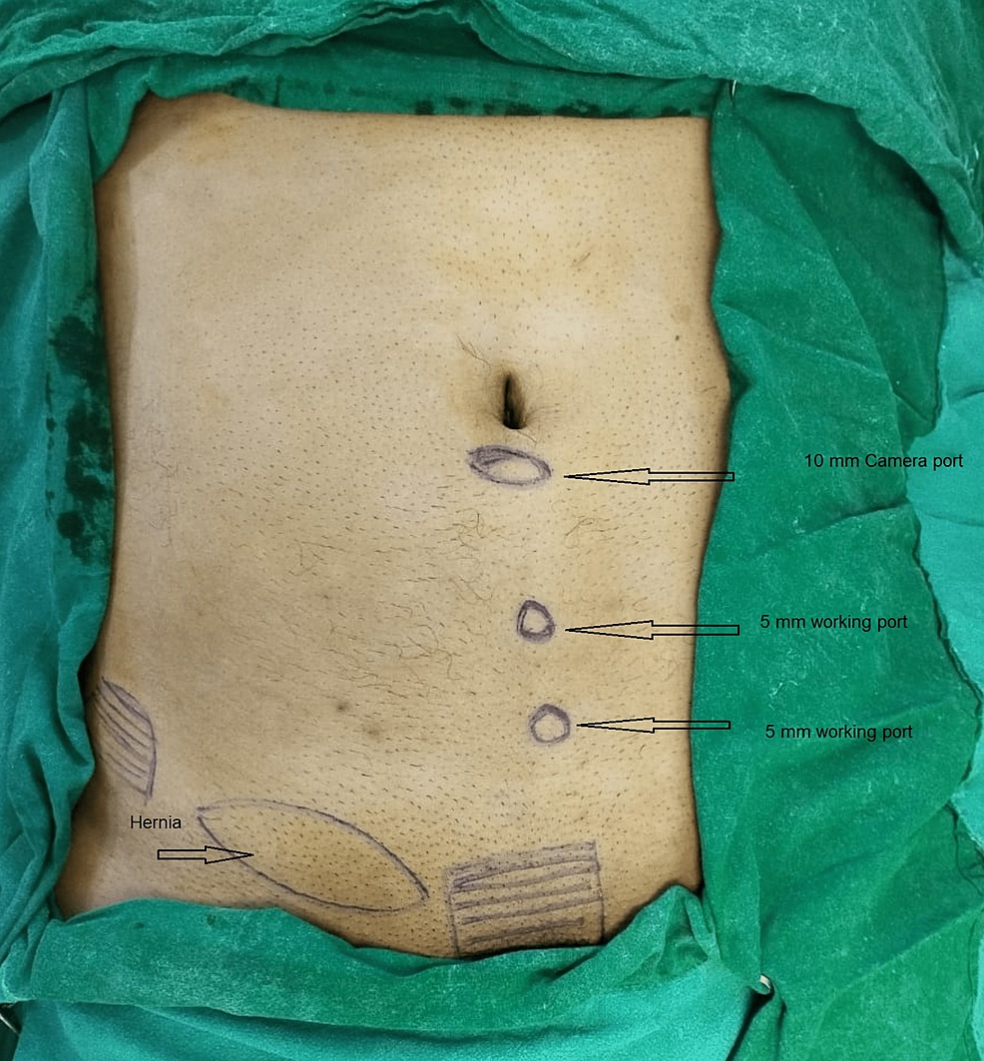
Port position for classical TEP TEP: totally extraperitoneal

**Figure 2:**
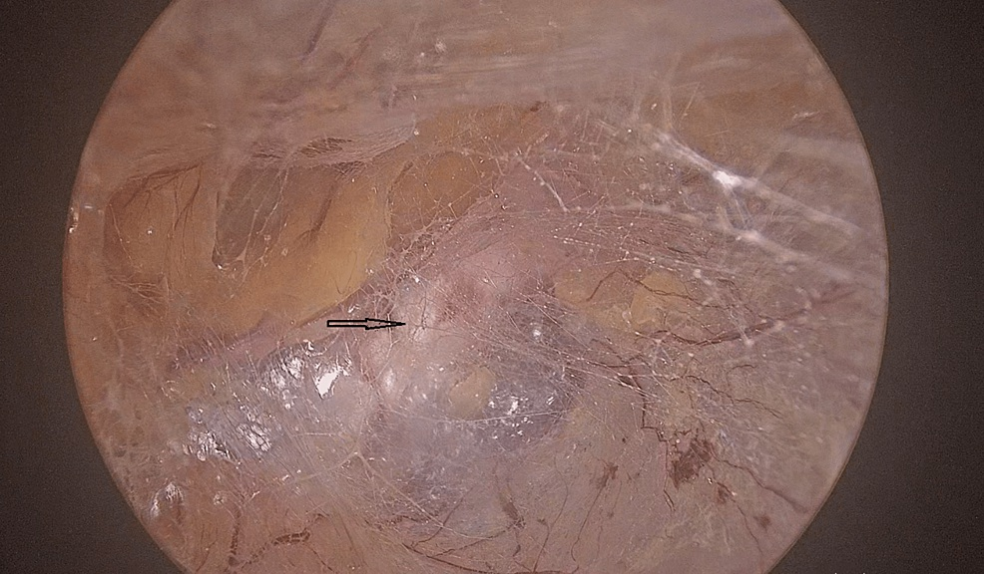
Light house sign

A 10-mm camera port was inserted in the midline just below the umbilicus. Blunt dissection was performed toward the pubic symphysis after insufflation of CO2. Two 5-mm working ports were placed in the midline (Figure 1). There was no crossover, and the lighthouse sign was appreciated (Figure 2). Dissection was carried out to create the space of Bogros and the cave of Retzius. Polypropylene mesh of appropriate size was kept in place over the myopectineal orifice of Fruchaud. An insufflation-desufflation test was carried out to check the position of the mesh. No tackers were used to fix the mesh, and the ports were closed with sutures. 

Operative steps for eTEP

**Figure 3:**
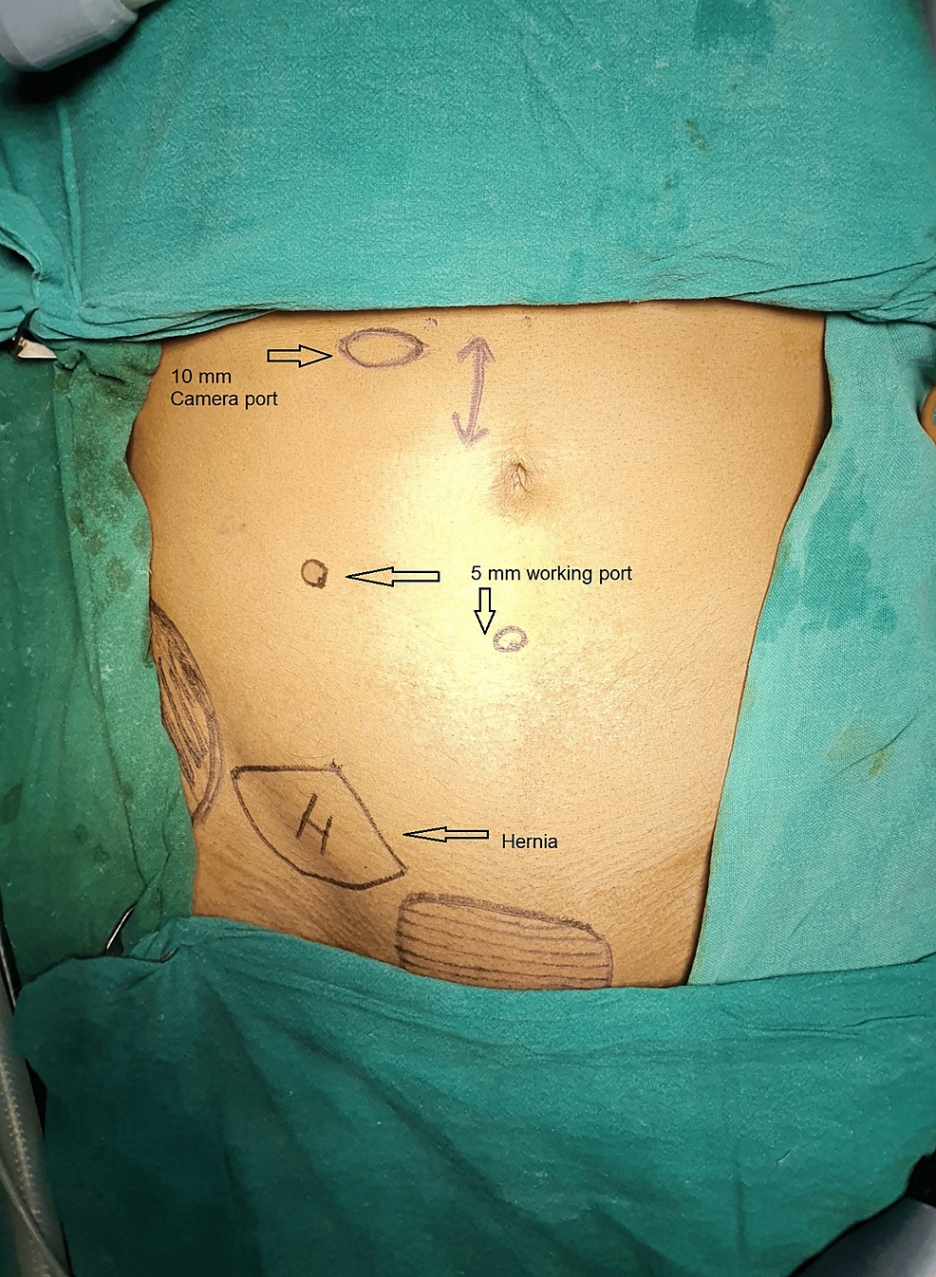
Port position for eTEP eTEP: extended totally extraperitoneal

**Figure 4:**
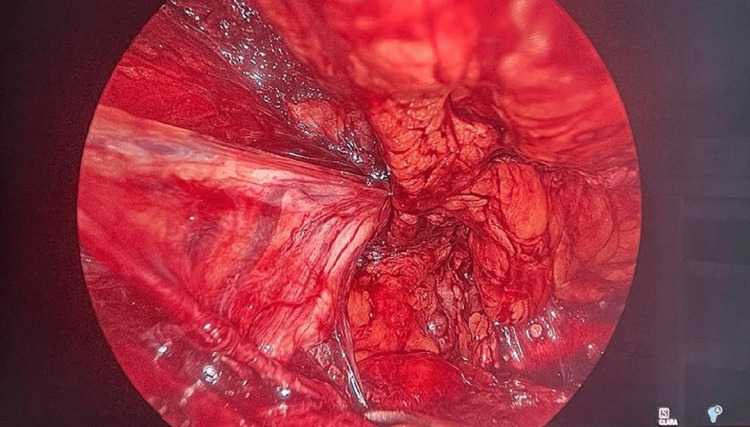
Cord structures in extraperitoneal space

A camera port was placed 2.5 cm cranially and laterally to the umbilical port to facilitate a wider surgical field. Blunt dissection similar to that used with the TEP approach, with 30° scope was performed toward the pubic symphysis after insufflation. Here, the two 5-mm working ports were placed bilaterally to achieve maximum triangulation (Figure 2 and Figure 3). The surgical space was maximized with the crossover technique involving separation of the linea alba to get to the other side (Figure 4) [2]. Dissection was carried out for parietalization of the cord, proceeding toward creating the space of Bogros and cave of Retzius. Mesh placement involved the book-fold technique of introducing the mesh into the preperitoneal space in both procedures. The mesh was introduced through the 10-mm port and placed over the myopectineal orifice of Fruchaud. Patients received similar perioperative management in both groups.

The incidences of accidental pneumoperitoneum were recorded for both groups. Major peritoneal rents necessitated conversion to TAPP repair. Major rents refers to the rents that could not be sutured together without tension to maintain the insufflated extraperitoneal space even with the use of a Veress needle. After minor rents were encountered, Veress needles could be used to manage accidental pneumoperitoneum and prevent conversion to TAPP repair. In both groups, this involved Veress needle insertion at Palmer’s point (3 cm below the left costal margin in the mid-clavicular line), as it is considered relatively free of adhesions [5]. All peritoneal rents were sutured closed to prevent visceral adhesions to nonbiologic mesh.

Statistical analysis

All p-values <0.05 were considered statistically significant. Analyses were conducted using SPSS Statistics for Windows, version 22.0 (IBM Corp., Armonk, NY, USA) [6-8].

## Results

Twenty-three out of 60 (38.3%) were 41-60 years of age. This coincides with the peak period of strenuous employment. The mean age was 48.28 years (Table 1). There was one woman in the TEP group after randomized allocation; all other participants were men.

**Table 1:** Demographic data: age, gender, site, type, duration of hernia TEP: totally extraperitoneal, eTEP: extended TEP

Characteristics	TEP	eTEP
Age (Years)		
21- 40	14 (46.7%)	6 (20.0%)
41-60	9 (30.0%)	14 (46.7%)
61-80	7 (23.3%)	9 (30.0%)
81-100	0 (0.0%)	1 (3.3%)
Gender		
Male	29 (96.7%)	30 (100.0%)
Female	1 (3.3%)	--
Site		
Right	13 (43.3%)	12 (40%)
Left	7 (23.3%)	10 (33.3%)
Bilateral	10 (33.3%)	8 (26.7)
Type		
Direct	8 (26.7%)	9 (30%)
Indirect	9 (30%)	9 (30%)
Both	13 (43.3%)	12 (40%)
Duration of hernia (months)	8.0 (3-24)	6.5 (2-18)

In the TEP group, 43.3% of the hernias were right-sided, 23.3% were left-sided, and 33.3% were bilateral. In the eTEP group, 40% were right-sided, 33.3% were left-sided, and 26.3% were bilateral (Table 1).

The median duration from hernia symptom onset in the TEP group was 8.0 months (range, 3-24 months). In eTEP group, the median symptom duration was 6.5 months (range, 2-18 months) (Table 1).

Direct, indirect, and mixed (direct and indirect) inguinal hernias comprised 26.7%, 30.0%, and 43.3% of the cases in the TEP group, respectively. In the eTEP group, 30.0%, 30.0%, and 40.0% were direct, indirect, and mixed hernias, respectively (Table 1).

**Figure 5:**
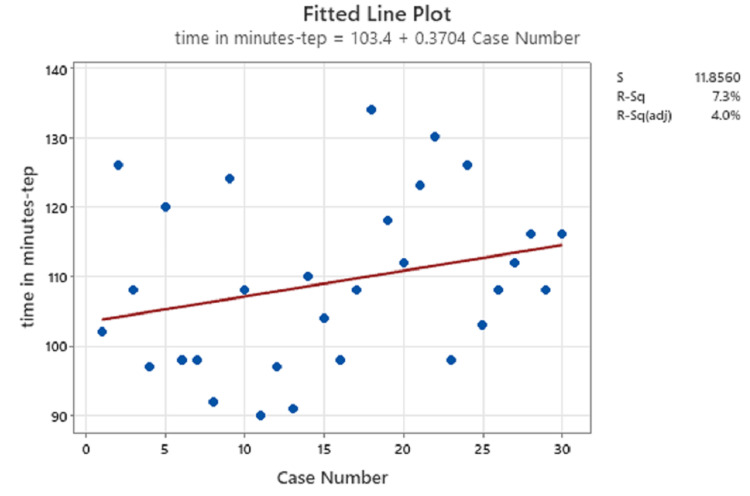
Linear regression of TEP cases TEP: totally extraperitoneal

**Figure 6:**
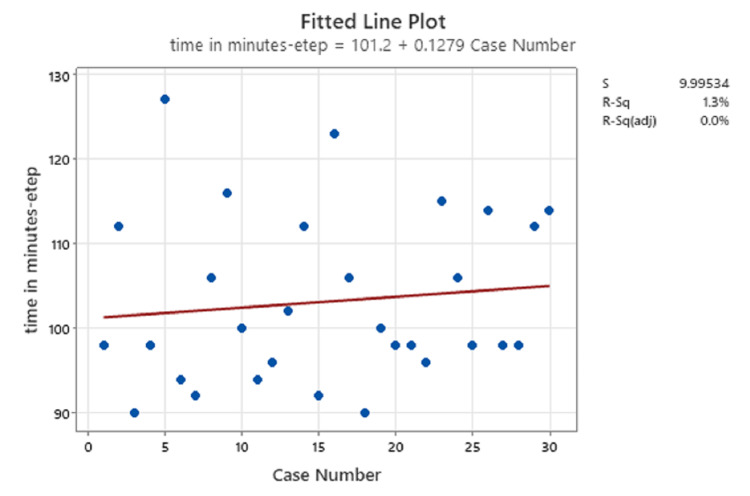
Linear regression of eTEP cases eTEP: extended totally extraperitoneal

The median operative time in the TEP group was 110 minutes (range, 90-134 minutes), compared with a median of 99 minutes in the eTEP group (P=0.031) (Table 2). Linear regression analysis revealed that there was no significant intergroup difference in this regard, owing to inclusion of bilateral and unilateral cases in the analysis (Figure 5 and Figure 6).

**Table 2:** Intraoperative and postoperative observations. TEP: totally extraperitoneal, eTEP: extended TEP

Observation	TEP	eTEP
Cord oedema	1 (3.4%)	0 (0.0%)
Seroma	1 (3.4%)	0 (0.0%)
Superficial wound infection	2 (6.7%)	0 (0.0%)
Persistent pain	1 (3.3%)	0 (0.0%)
Accidental pneumoperitoneum	3 (10.0%)	2 (6.7%)
Median duration of surgery (minutes)	110 (90-134)	99 (90-127)
Median hospital stay (days)	2 (1-4)	2 (2-4)
Median time to return to daily activities (days)	4 (3-6)	3 (2-5)

There were no vascular or visceral injuries in either group, and no cases of laparoscopic hernia repair were converted to open repair.

In the TEP and eTEP groups 10.0% and 6.7% (P=0.64) of the procedures involved accidental pneumoperitoneum, respectively, all of which were managed effectively with Veress needles (Table 2).

Pain was evaluated and analyzed based on a visual analog scale, and all patients were treated with oral paracetamol for postoperative pain as needed. The median duration of pain in both groups was two days (Table 2).

In the TEP group, one patient (3.4%) had postoperative cord edema. Clinical findings were confirmed on local USG. Patients were managed conservatively with oral paracetamol. No cord edema was noted in the eTEP group. One TEP inguinal hernia repair (3.4%) was complicated by postoperative seroma formation, and this was managed conservatively. Two surgical site infections (3.3%) were managed conservatively in the TEP group. Persistent pain affected one patient (3.4%) in the TEP group but none in the eTEP group. There were no mesh infections in either group (Table 2).

The median duration of hospital stay in both groups was two days, and the median times to return to daily activities (regular work) were four and three days, respectively, in the TEP and eTEP groups (Table 2).

## Discussion

Laparoscopic TEP repair of inguinal hernias is a relatively new modality in minimally invasive surgery. However, it is associated with some difficulties, such as a limited operative view, ergonomic difficulties for large inguinal hernias, and a higher risk of entering the peritoneal cavity [9]. Traditional TEP repair was revolutionized by Prof. Daes, who introduced the eTEP approach in 2012. It shortened the learning curve for aspiring endoscopic surgeons by allowing flexible port positions, the crossover technique for an extended view, and a lower risk of pneumoperitoneum, promising reproducible results and improving the ergonomics of the procedure.

Twenty-three out of 60 (38.3%), were in the 41-60-year age group, and the mean age was 48.28 years. This coincides with the peak period of the worker’s heavy job especially in developing countries. The mean age was 49.34 in the study conducted by Prakhar et al. in India [10].

In the present study, the median time taken for laparoscopic TEP repair was 110 minutes (range, 90-134), compared with 99 minutes (range, 90-127) for eTEP repair. Linear regression analysis revealed that there was no significant intergroup difference in this regard, owing to the inclusion of bilateral and unilateral cases in the analysis (Figure 5 and Figure 6). However, when considering only unilateral hernias, the median operative time was significantly higher in the TEP repair group compared with the eTEP group (P=0.031). In the study conducted by Prakhar et al., the mean operative time was 113.08±63.30 [10].

Initially, due to the difficult learning curve associated with advanced minimally invasive techniques of hernia repair, accidental rents in the peritoneum are anticipated. The rents lead to escape of CO2 into the peritoneal cavity and cause ballooning of the peritoneum which obscures the working field as well reducing it. The primary interest of the surgeon is always to safeguard important structures especially vascular which at times may lead to accidental rents in the peritoneum at the cost of safeguarding important structures. Ten percent of the cases in the TEP group required the use of Veress needles to manage pneumoperitoneum, compared with 6.7% in the eTEP group. The intergroup difference was not statistically significant (P=0.6404). Sudarshan et al. reported the use of Veress needles in the management of pneumoperitoneum in TEP and eTEP inguinal hernia repair [11].

In the present study, the median duration of postoperative pain among the cases studied in the TEP and eTEP groups was two days in both groups. Decrease in postoperative pain is one of the important advantages of minimally invasive surgery. It also helps to reduce the duration of hospital stay of the patient. The shorter the duration of hospitalization associated with laparoscopic hernia repair, the lower the incidence of hospital-acquired infection [12]. The median duration of hospital stay in both groups was two days. In the retrospective single-center study conducted by Baig et al., the median duration of hospital stay was three days [9].

We found that 3.4% of the cases in the TEP group had postoperative seroma formation. None of the patients who underwent eTEP repair experienced postoperative seromas. Baig et al. observed a postoperative seroma incidence of 4.7% [9]. Incidence of seroma can be reduced with meticulous dissection techniques.

In the TEP group, 3.3% of patients had evidence of infection at the 10-mm trocar site. The incidence of surgical site infection was 2.94% in the study conducted by Prakhar et al. in India [10]. Aseptic precautions before surgery and comprehensive postoperative care can reduce incidences of surgical site infection (SSI).

The median postoperative intervals before returning to daily activities were four days in the TEP group and three days in the eTEP group. In the study by Ngo et al., the mean interval that elapsed before patients returned to daily activities was four days [13]. Minimally invasive techniques help patients to return to daily activities (work) early and hence can be considered economically more appealing.

This study was conducted at a single center with procedures carried out by a single surgeon; these were among the study’s limitations. A study incorporating multiple centers and surgeons, along with a larger sample size, would help further validate our findings regarding the advantages and disadvantages of these methods. A more robust multicenter study design (randomized controlled trial, for example) would help us further evaluate the superiority of one procedure over the other.

## Conclusions

Laparoscopic hernia repair via the eTEP approach has a shorter learning curve compared with the TEP approach due to port flexibility and better ergonomics. The primary aim of the study was to compare incidence of accidental pneumoperitoneum associated with TEP and eTEP hernia repair. The study concludes that there is no statistical difference between the two procedures in terms of incidence of accidental pneumoperitoneum. Veress needles can be used to manage peritoneal rents causing pneumoperitoneum, especially during the early learning stages. The eTEP approach is advantageous over the TEP approach in terms of operative time, owing to the shorter learning curve, wider-angle view, wider range of motion for instrument manipulation, and ergonomically superior operative experience. The advantages of the TEP approach include that it involves less dissection of the extraperitoneal space, leaving more area for redo surgeries. The limited space is a disadvantage of the TEP approach in that it means limited room for dissection, a higher risk of accidental pneumoperitoneum, and a longer learning curve.

This study was conducted to highlight differences in benefits and outcomes between these procedures. However, most of these shortcomings can be minimized once the surgeon gains enough experience and enters the plateau phase of the learning curve.
